# A Rare Case of a Left Ventricular Pseudoaneurysm With Lateral Wall Rupture

**DOI:** 10.7759/cureus.22909

**Published:** 2022-03-07

**Authors:** Abdullah Al Lawati, Wijdan Al-Hadhrami, Fatma Al Hosni, Meetham Al Lawati, Emad Elseragy, Srinivasa Sirasanagandla, Adil Al Lawati, Mustafa Al-Attraqchi, Rashid Saif AL Umairi

**Affiliations:** 1 College of Medicine and Health Sciences, Sultan Qaboos University, Muscat, OMN; 2 Cardiothoracic Surgery, The Royal Hospital, Muscat, OMN; 3 Department of Human and Clinical Anatomy, College of Medicine and Health Sciences, Sultan Qaboos University, Muscat, OMN; 4 Radiology, The Royal Hospital, Muscat, OMN

**Keywords:** echocardiogram, pericardial effusion, surgical emergency scenarios, myocardial infarction complication, lateral wall rupture, left ventricular pseudoaneurysm

## Abstract

In this report, we present a case of a 51-year-old male patient with a left ventricular (LV) pseudoaneurysm and a ruptured lateral wall due to a previous myocardial infarction. This patient was referred to the Coronary Care Unit with a past history of acute coronary syndrome of two months. He presented with palpitations and acute pulmonary edema upon admission. Color Doppler detected a ruptured lateral ventricular wall, and an echocardiogram confirmed the presence of a lateral ventricular wall pseudoaneurysm. Emergency LV aneurysmal rupture repair surgery was performed on this patient, and the postoperative findings were stable till discharge.

## Introduction

A left ventricular (LV) pseudoaneurysm is formed when a free myocardial wall rupture is contained by the surrounding pericardium and scar tissue [1]. A pseudoaneurysm differs from true aneurysms as it contains no myocardium or pericardium. LV pseudoaneurysms are very rare, with an incidence of 0.23% [2]. Myocardial infarction (MI) and surgeries are the most common etiologies, with the former accounting for 55% of the cases [1]. Common symptoms include dyspnea, chest pain, and congestive heart failure [3]. In this report, we present an unusual case of LV pseudoaneurysm with lateral wall rupture. This case was unusual as the initial presenting symptoms were not indicative of a severe cardiac abnormality, when, in fact, there was a massive infarction of the lateral wall that could have killed the patient due to arrhythmia or aneurysm rupture.

## Case presentation

**Figure 1:**
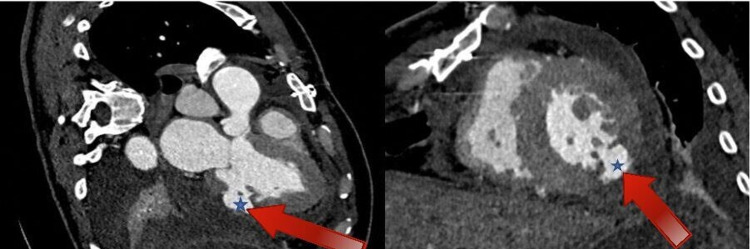
Cardiac CTA showing a contrast-filled outpouch related to the inferolateral wall of the LV associated with a pericardial effusion in keeping with a pseudoaneurysm (red arrow, blue star). CTA, computed tomography angiography; LV, left ventricle

A 51-year-old patient with a previous medical history of diabetes mellitus and hypertension was referred to our tertiary hospital and admitted with acute pulmonary edema and palpitations. He was an active smoker, and was diagnosed with acute coronary syndrome and put on aspirin two months prior to his visit. Lab investigations showed that the troponin level was elevated at 0.169 ng/mL (normal: <0.040 ng/mL) [4]. Other lab values included glomerular filtration rate (GFR), which was 58 mL/min, and hemoglobin, which was 14 g/dL. An echocardiogram showed an ejection fraction of 55%, non-dilated left ventricle, inferior and inferoposterior hypokinesis, normal left atrium/aortic root size, mild mitral regurgitation, and ruptured lateral wall of the left ventricle which contained a large pseudoaneurysm, compressing the left atrium and mitral valve. The coronary angiogram showed a large and dominant right coronary artery, with 60% proximal and 30% distal lesions. The left main coronary artery showed a 20% mid-shift lesion, and the second obtuse marginal artery of the left circumflex artery showed a subtotal proximal occlusion after the first obtuse marginal artery, while the other coronary arteries were normal. Echocardiogram was repeated, which confirmed the presence of a pseudoaneurysm in the lateral wall of the left ventricle with a large sac and a defect in the inferolateral portion of the left ventricle wall. There were multiple defects that collectively measured to 10-12 mm, whereas the sac measured 5-7 cm. The EuroSCORE, a prognostic model that predicts the risk of mortality from a surgery, was low at 1.76% (reference range for low risk is 2-4%) [5]. In addition, a cardiac computed tomography angiography (CTA) was performed one week pre-operatively, which showed pericardial effusion with dense fluid around the anterior and lateral LV wall with features in keeping with hematoma, as demonstrated in Figure 1. The maximum depth was 21 mm. Coronary artery bypass graft surgery (CABG) was not performed due to the circumflex artery lesion. Thus, the patient was scheduled for emergency surgery to repair the LV aneurysm. Another echocardiogram was performed post-operatively, which showed the presence of no aneurysm. Upon a follow-up period of three months, the patient was still doing well with no complaints.

## Discussion

A life-threatening complication of MI is rupture of the LV free wall, occurring in roughly 4% of patients with infarcts and around 23% of those suffering fatal infarcts. Free wall rupture is rarely contained by the overlying, adherent pericardium, resulting in a pseudoaneurysm or false aneurysm of the left ventricle [3]. Thus, unlike a true aneurysm, LV pseudoaneurysm contains no endocardium or myocardium.

LV pseudoaneurysms have been found to mostly occur in the posteroinferior wall and in basal segments rather than in apical segments. The paucity of anterior LV pseudoaneurysms could be explained by the fact that an anterior rupture is more likely to cause a hemopericardium and death than a posterior rupture. Moreover, because most hospitalized patients remain in a recumbent position, an inflammatory reaction of the posterior pericardium might cause pericardial adhesions and the establishment of a posterior LV pseudoaneurysm [6].

The majority of LV pseudoaneurysms develop after MI or cardiothoracic surgery. In a systematic literature review of 290 patients, MI (55%), surgery (33%), and trauma (7%) were the top three associations. LV pseudoaneurysms pose a significant risk of rupture, which is far higher than that of a true aneurysm. Congestive heart failure, chest pain, and dyspnea were the most commonly reported symptoms in patients with LV pseudoaneurysm, according to one systematic literature review. In addition, the clinical presentation of patients with LV pseudoaneurysms is varied. According to the systematic literature evaluation, more than 10% of patients (n = 290) were asymptomatic [7]. Furthermore, 48% (n = 52) of the cases in a Mayo Clinic case series on cardiac pseudoaneurysms were asymptomatic [8].

Noninvasive imaging modalities, including echocardiography, CTA, and cardiac magnetic resonance imaging (MRI), are the investigations of choice for diagnosing LV pseudoaneurysm [9]. Color flow imaging improved diagnostic accuracy by allowing flow in and out of the aneurysm as well as within the pericardial space to be detected, even when the pulsed waves and continuous Doppler were ineffective [10]. When a rapid diagnosis is required, transesophageal echocardiography can be performed. However, it is limited by the need for competent operators, which will be difficult for a critically ill patient. Lately, contrast echocardiography has been proven to be particularly useful when diagnostic uncertainty persists, and the acoustic windows are inadequate. Contrast should show in the pericardial region, with contrast in the left ventricle cavity lining the left ventricle wall. This will allow for a quicker diagnosis, and there has been some emerging evidence of using contrast echocardiography to confirm the diagnosis in the emergency room, allowing for a quick operation [11]. It can be challenging to differentiate between LV pseudoaneurysm and true aneurysm. On echocardiography, one technique to determine this is to compare the diameter of the aneurysm's neck/orifice to its maximum diameter. Pseudoaneurysms tend to have a narrow orifice and therefore have a small neck/orifice to maximum diameter ratio, which is usually less than 0.50, as compared to true aneurysms, which have a broad orifice [12]. There is growing evidence that MRIs can distinguish between true aneurysms and pseudoaneurysms [13].

LV pseudoaneurysm has a strong indication for surgery since untreated pseudoaneurysms have a 30%-45% chance of rupturing [14]. Unless the surgical risk is excessive, the general consensus is that surgery should be performed on all patients as soon as the diagnosis is made. Due to the increased risk of rupture, if a pseudoaneurysm is detected within two to three months of an MI, immediate surgery should be performed. The necessity and urgency of surgery depend on the symptoms if the diagnosis is made years after an MI. Conservative treatment is used when the LV pseudoaneurysm is less than 3 cm and is discovered incidentally. In symptomatic patients, resection combined with bypass grafting is the preferred treatment [15]. Moreover, for individuals with high surgical risk, the proper treatment of choice is the percutaneous repair of LV pseudoaneurysm.

## Conclusions

LV pseudoaneurysms are very rare and often present with dyspnea, chest pain, and congestive heart failure. MIs are the most common cause of LV pseudoaneurysm followed by cardiothoracic surgery. Multiple diagnostic tools are available, such as echocardiography, CTA, and cardiac MRI. LV pseudoaneurysms are strong indications for surgery. New techniques need to be developed to treat LV pseudoaneurysm as current surgeries carry a reasonable risk.
